# Perioperative H-FABP and Its Association with Postoperative Morbidity Following Pancreatic Resection: A Prospective Observational Study

**DOI:** 10.3390/medicina62091745

**Published:** 2026-09-10

**Authors:** Danaja Plevel, Ana Kalamutova, Aleš Jerin, Mihajlo Đokić, Aleš Tomažič, Blaž Trotovšek, Miha Petrič

**Affiliations:** 1 Department of Abdominal Surgery, University Medical Center Ljubljana, 1000 Ljubljana, Slovenia; danaja.plevel@kclj.si (D.P.);; 2 Faculty of Medicine, University of Ljubljana, 1000 Ljubljana, Slovenia; 3 Institute of Clinical Chemistry and Biochemistry, University Medical Center Ljubljana, 1000 Ljubljana, Slovenia; 4 Faculty of Pharmacy, University of Ljubljana, 1000 Ljubljana, Slovenia

**Keywords:** heart-type fatty acid binding protein, pancreatic surgery, complications, postoperative pancreatic fistula, biomolecular marker

## Abstract

*Background and Objectives*: Postoperative morbidity presents a significant challenge in pancreatic surgery. Biomarkers that enable early detection of complications could enhance postoperative monitoring and allow for timely intervention. This study aimed to assess whether perioperative concentrations of heart-type fatty acid–binding protein (H-FABP) are linked to severe postoperative complications and pancreas-specific morbidity following pancreatic resection. *Materials and Methods*: Serum H-FABP levels were measured preoperatively and on postoperative days 2, 4, and 6. *Results*: Among the 53 patients (33 women [62.3%]) included in the final analysis, those with malignant disease exhibited significantly higher preoperative H-FABP concentrations (median, 1320 vs. 630 ng/L; *p* = 0.015), as did patients classified as ASA III (median, 1309 vs. 620 ng/L; *p* = 0.025) and aged 70 years or older (median = 1431 vs. 550 ng/L; *p* < 0.001). Elevated postoperative H-FABP levels were associated with severe complications, with notable increases observed on postoperative day (POD) 2 (*p* = 0.026), POD4 (*p* = 0.022), and POD6 (*p* = 0.015). Exploratory ROC analysis indicated moderate discrimination for severe complications (AUC, 0.70, 0.71, and 0.73, respectively). No significant differences in postoperative H-FABP levels were found among patients with clinically relevant postoperative pancreatic fistula, postpancreatectomy acute pancreatitis and postpancreatectomy hemorrhage. *Conclusions*: Exploratory comparisons at individual time points showed higher postoperative concentrations of H-FABP in patients with severe complications; however, the main longitudinal analysis using a mixed-effects model did not reveal a statistically significant group effect or an interaction between group and time. Larger prospective multicenter studies are required to validate its clinical utility.

## 1. Introduction

Pancreatic resection is one of the most complex procedures in abdominal surgery. Advances in surgical techniques and perioperative management have resulted in an acceptable mortality rate of up to 3% in large series [1]. However, it remains associated with significant postoperative morbidity. Recent series report overall complication rates between 30% and 60%, with clinically relevant postoperative pancreatic fistula (CR-POPF), delayed gastric emptying (DGE), and postpancreatectomy hemorrhage (PPH) representing the main reasons of prolonged hospitalization and higher cost [2,3].

Numerous risk stratification factors have been developed to predict the incidence of postoperative complications. The International Study Group of Pancreatic Surgery (ISGPS) categorizes risks for POPF from A (low risk) to D (high risk) based on the diameter of the pancreatic duct and the texture of the pancreatic gland [4]. Despite surgical bias in defining the type of texture, the Fistula Risk Score accurately predicts subsequent CR-POPF. Other influencing factors for prediction of postoperative complications include patient-related variables (age, comorbidity burden, and American Society of Anesthesiologists (ASA) classification), and procedure-related characteristics [5]. However, these models rely primarily on static anatomical or clinical variables and do not capture the dynamic physiologic stress response associated with major surgery. Despite the growing interest in perioperative biomarkers, most currently used risk stratification models in pancreatic surgery rely primarily on anatomical and technical variables. While these models are useful for predicting pancreas-specific complications, they provide limited information regarding the overall patient’s state and their vulnerability.

Currently, the most effective strategy to reduce mortality involves early identification and minimally invasive management of complications following pancreatic resection, which significantly enhances clinical outcomes compared to standard care. Implementation of structured postoperative management algorithms has been associated with approximately a 50% reduction in 90-day mortality [6]. A biomarker reflecting real-time cellular injury and stress could further improve perioperative monitoring and facilitate early detection of clinical deterioration.

Several biomarkers have been investigated to improve early postoperative risk stratification after pancreatic resection. Among pancreas-specific markers, drain amylase remains the cornerstone for the early identification and exclusion of clinically relevant postoperative pancreatic fistula (CR-POPF), while drain lipase has also demonstrated promising diagnostic performance [7,8]. Systemic inflammatory biomarkers, including C-reactive protein (CRP) and procalcitonin (PCT), have likewise been associated with postoperative infectious complications and CR-POPF, although their diagnostic accuracy varies across studies and optimal cut-off values remain inconsistent [9,10]. Consequently, current postoperative management increasingly relies on a multimodal approach that combines clinical assessment with biochemical markers rather than any single biomarker alone [7,11].

H-FABP is a small cytosolic protein, approximately 15 kDa in size, that is abundantly expressed in cardiomyocytes, where it facilitates intracellular fatty acid transport to mitochondria for β-oxidation [12]. Because of its cytoplasmic localization and low molecular weight, H-FABP is rapidly released into circulation after disruption of cellular membrane integrity. Plasma H-FABP concentrations increase within 1 to 3 h after myocardial injury and peak earlier than cardiac troponins, making it an early marker of myocardial damage [13,14].

Beyond acute coronary syndromes, H-FABP has demonstrated prognostic relevance in broader cardiovascular and perioperative contexts. Perioperative myocardial injury after noncardiac surgery (MINS) is associated with increased 30-day mortality [15]. Even minor postoperative elevations in cardiac biomarkers have been associated with worse outcomes [15]. Given that H-FABP is released earlier than troponins and may detect subclinical myocardial strain, it has been proposed as a sensitive marker of perioperative cardiovascular stress and may reflect early cardiomyocyte membrane permeability changes triggered by surgical stress [16,17]. Therefore, H-FABP was selected as a biologically plausible biomarker for exploratory evaluation in this setting, rather than troponins that are the reference biomarker for myocardial infarction with irreversible cardiomyocyte necrosis [17].

Major abdominal surgery is associated with a substantial risk of perioperative myocardial injury and other postoperative complications, which remain important contributors to short- and long-term morbidity and mortality [17,18]. Although H-FABP has been investigated in cardiovascular disease, critical illness, and perioperative myocardial injury after noncardiac surgery, its role in major abdominal surgery—and specifically in pancreatic resection—has not yet been systematically studied.

In addition to cardiac-specific injury, H-FABP may also reflect global metabolic stress. Fatty acid metabolism is central to myocardial energy production, and systemic inflammatory states are known to alter substrate utilization and mitochondrial efficiency [12,19]. Pancreatic malignancy itself is associated with systemic inflammation, increased metabolism, and cancer-related cachexia, all of which may influence myocardial state and baseline H-FABP concentrations. Thus, perioperative H-FABP concentrations may be influenced by systemic and patient-related factors in addition to cardiac injury.

The primary aim of this exploratory study was to investigate the association between perioperative H-FABP concentrations and severe postoperative morbidity following pancreatic resection. Secondary aims were to explore associations between H-FABP concentrations and pancreas-specific postoperative complications and preoperative clinical characteristics.

## 2. Materials and Methods

We conducted a prospective observational cohort study. We adhered to the STROBE checklist guidelines to ensure accurate and comprehensive reporting.

The study was conducted at the Department of Abdominal Surgery, University Medical Center Ljubljana, from July 2023 to October 2024. We included 157 consecutive patients. Inclusion criteria were adult patients (age ≥ 18 years) who were candidates for any kind of pancreatic resection. At the end of the study period, 45 patients were excluded because of the exploratory nature of their surgeries or the performance of total pancreatectomy, which eliminated the possibility of developing a pancreatic fistula. A total of 112 eligible patients were evaluated for the completeness of the four sample sets, resulting in the exclusion of 59 patients with incomplete data. For the remaining 53 patients, postoperative complications were prospectively documented during initial hospitalization. Post-discharge events, including hospital readmissions and subsequent evaluations, were assessed by reviewing the hospital database. Ninety-day mortality was determined using data from hospital databases and the national registry.

Patients were categorized based on age into two groups: <70 years and ≥70 years. Based on diagnosis, we categorized the patients into two groups: malignant (adenocarcinoma, neuroendocrine tumors) and non-malignant histopathology (intraductal papillary mucinous neoplasm (IPMN), mucinous cystic neoplasm (MCN), serous cystic neoplasm (SCN), and others). Demographic data, including age, sex, and ASA score, and postoperative outcomes such as CR-POPF, postpancreatectomy hemorrhage (PPH), postpancreatectomy acute pancreatitis (PPAP), delayed gastric emptying (DGE), bile leak (BL) and 90-day mortality were collected in accordance with the study protocol. The detailed study protocol is provided in Appendix A. Postoperative complications were classified using the Clavien–Dindo classification system, with severe postoperative complications defined as Clavien–Dindo grade ≥ III [20]. Postoperative complications, such as CR-POPF, PPH, PPAP, and DGE were classified according to the corresponding ISGPS definitions [2,21,22,23]. BL was classified according to the grading system established by the International Study Group of Liver Surgery [24].

Venous blood samples for serum FABP analysis were obtained on the day of surgery upon admission to the hospital ward and on postoperative days (POD) 2, 4 and 6. Blood samples were collected without additive. Serum was separated after centrifugation (1500× *g* for 10 min) and aliquots were stored at −20 °C until analyses. H-FABP analysis was performed only in patients for whom a complete set of four serum samples (preoperatively and on POD2, POD4, and POD6) had been successfully collected. The specific missing sampling time point was not systematically recorded for all excluded patients, and H-FABP assays were not performed in patients without a complete four-sample set.

The concentrations of H-FABP were measured using an enzyme-linked immunosorbent assay (ELISA) (Cusa Biotechnology Ltd., Houston, TX, USA). The analytical sensitivity, established by the manufacturer as the mean value of 20 replicate measurements, was 19.5 ng/L (catalog number.: 1C-CSB-E09185H-96; LOT L08066882). Precision performance was assessed using three quality control samples with known concentrations, each analyzed in 20 replicates. The intra-assay and inter-assay imprecision, expressed as coefficients of variation (CVs), were <8% and <10%, respectively.

H-FABP measurements were performed by laboratory personnel blinded to the patients’ clinical data and postoperative outcomes. Outcome assessors were similarly blinded to H-FABP concentrations during postoperative data collection and complication assessment.

The study was conducted in accordance with the Declaration of Helsinki and approved by the National Medical Ethics Committee of the Republic of Slovenia (0120-493/2021/5, 9 June 2022).

### Sample Size and Statistical Analysis

**Figure 1:**
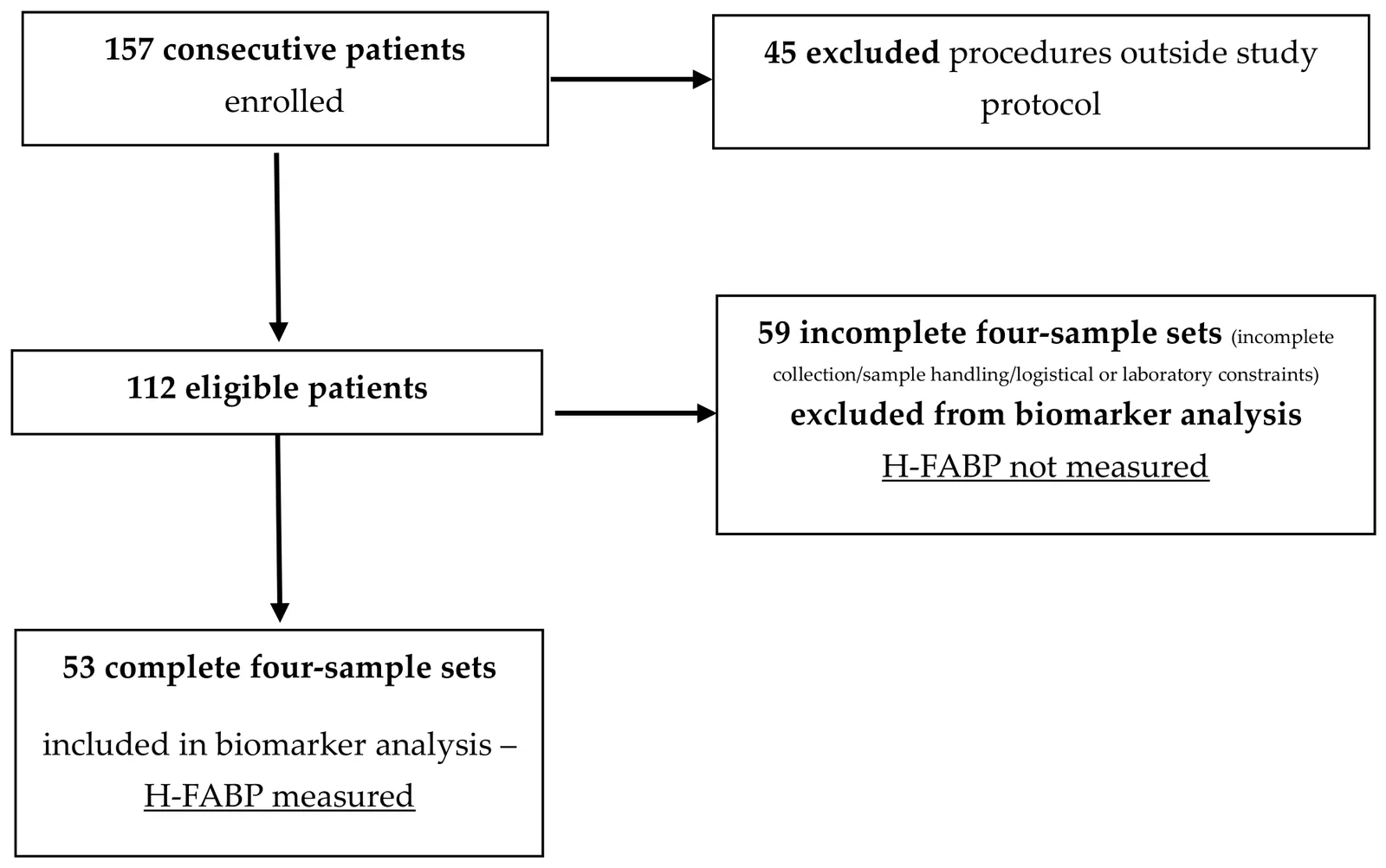
Flow diagram of patient selection and inclusion in the H-FABP analysis.

No a priori sample-size calculation was performed, as this cohort included all consecutive patients who met the eligibility criteria during the study period. Of the 157 enrolled patients, 45 were excluded because they underwent procedures outside the study protocol (e.g., exploratory laparotomy, biopsy, biliodigestive procedures, or total pancreatectomy). Among the remaining 112 eligible patients, 59 did not have a complete set of four scheduled serum samples because of incomplete sample collection, sample-handling issues, or logistical constraints. Consequently, 53 patients with complete sample sets were included in the final analysis. The patient selection process is summarized in Figure 1. To assess potential bias, we compared the 59 excluded patients with the 53 included patients in terms of gender, ASA classification, malignancy, type of surgical procedure, and postoperative outcomes.

The primary clinical outcome was severe postoperative morbidity, defined as Clavien–Dindo grade III or higher. For patients experiencing multiple complications, the highest Clavien–Dindo grade reached during the postoperative course was used for severity classification.

Statistical analyses were performed using IBM SPSS Statistics version 29 (IBM Corp., Armonk, NY, USA), Jamovi (version 2.3), and R (version 2025.05.1+513). All tests were two-tailed, with statistical significance defined as *p* < 0.05. The distribution of continuous variables was assessed using the Shapiro–Wilk test. Because FABP concentrations and other clinical variables were not normally distributed, between-group comparisons were conducted using the Mann–Whitney U test. Effect sizes for Mann–Whitney U tests were calculated as rank-biserial correlation (r), computed as r = 1 − (2U)/(n_1_n_2_), where U is the Mann–Whitney U statistic and n_1_, n_2_ are the sample sizes of the two groups. Time-point-specific comparisons between patients with and without severe morbidity were performed using the Mann–Whitney U test and are presented as exploratory descriptive analyses. These comparisons were not considered independent confirmatory tests.

Because postoperative H-FABP concentrations were repeatedly measured within the same patients on POD2, POD4, and POD6, the longitudinal association between H-FABP and severe postoperative morbidity was assessed using a linear mixed-effects model. H-FABP concentrations were transformed using log (H-FABP + 1) because of their right-skewed distribution. Time, severe-complication status, and the time × severe-complication interaction were included as fixed effects, with a patient-specific random intercept to account for within-patient correlation. No single postoperative time point was prospectively designated as the primary time point; therefore, the mixed-effects model was considered the principal analysis of serial postoperative H-FABP measurements.

Categorical variables were compared using Fisher’s exact test due to small, expected cell counts, and odds ratios with 95% confidence intervals were reported where appropriate. Univariable binomial logistic regression models were used to explore associations between H-FABP concentrations and individual postoperative outcomes. Model performance was assessed using McFadden’s R2 and the AUC. Receiver operating characteristic (ROC) curve analysis was performed as an exploratory assessment of the ability of H-FABP concentrations to discriminate between patients with and without severe postoperative morbidity. Areas under the ROC curve (AUCs) with 95% confidence intervals were reported. Given the small number of severe events and absence of an independent validation cohort, data-derived optimal cut-off values were not calculated. For concentrations below the assays lower limit of detection, which corresponds to the manufacturer reported analytical sensitivity, values were imputed using LOD/2. Values below the threshold level were absent in all samples prior to surgery. However, they were observed in one patient in the sample group on POD2 and POD4, and in eight patients in the sample group on POD6. All analyses were based on complete-case data. Given the exploratory nature of the study and the multiple comparisons across outcomes and time points, no formal adjustment for multiplicity was applied. Accordingly, *p*-values from secondary and time-point-specific analyses should be interpreted as exploratory.

## 3. Results

### 3.1. Baseline Characteristics

A total of 157 consecutive patients were enrolled, of whom 45 were excluded because they underwent procedures outside the study protocol. Of the remaining 112 eligible patients, 59 were excluded from the analysis because a complete four-sample set was unavailable, leaving 53 patients in the final analysis. The analyzed cohort comprised 33 women (62.3%) and 20 men (37.7%), with a mean age of 68.2 ± 8.7 years. Twenty-five patients (47.2%) were aged < 70 years and 28 (52.8%) were aged ≥ 70 years. Baseline characteristics of the analyzed and excluded cohorts are presented in Table 1.

There were no significant differences in ASA score, age, or etiology of pancreatic disease. No statistically significant differences in the distribution of procedures were observed between the two groups. In the study group, no 90-day mortality was observed, whereas the excluded group had a mortality rate of 5% (3 out of 59). However, this difference was not statistically significant (*p* = 0.24). All three deaths in the excluded cohort occurred after POD6.

### 3.2. Clinical Characteristics and Overall Postoperative Complications

In the analyzed cohort, 26 patients underwent open surgery, 11 underwent laparoscopic surgery, and 16 underwent robotic surgery. No significant differences were observed in the proportion of patients who experienced postoperative complications among the open, laparoscopic, and robotic approaches. The proportion of patients with postoperative complications was comparable between those with non-malignant disease (70.6%) and those with malignant disease (72.2%), with this association not reaching statistical significance (*p* = 0.902; Cramér’s V [V] = 0.017). Postoperative complications occurred in 13 of 19 patients with ASA 2 (68.4%) and in 25 of 34 patients with ASA 3 (73.5%), which was not statistically significant (*p* = 0.692; V = 0.054). Complications were observed in 6 of 11 patients without comorbidities (54.5%) and in 32 of 42 patients with at least one comorbidity (76.2%). Although overall complications were more frequent among patients with comorbidities, this association was not statistically significant (*p* = 0.258; V = 0.195). There was no significant association between age group and postoperative complications (*p* = 0.761). The complication rates were similar in patients < 70 years old (68.0%) and those aged 70 years or older (75.0%). The strength of the association was very small (V = 0.078). The odds of developing a postoperative complication were 1.41 times higher in patients aged ≥70 years than in younger patients; however, this effect was not statistically significant, and the confidence interval was wide (95% CI = 0.43–4.68).

### 3.3. Preoperative Serum H-FABP and Malignant vs. Non-Malignant Etiology

A significant difference was observed in preoperative H-FABP concentrations between patients with non-malignant and malignant histopathology. Individuals with malignant disease had markedly higher H-FABP levels (median = 1320 ng/L, interquartile range [IQR] = 817) compared with those with non-malignant pathology (median = 630 ng/L, IQR = 884), and this difference was statistically significant (Mann–Whitney U statistic [U] = 177.50, *p* = 0.015). The effect size was moderate (r = 0.420).

### 3.4. Preoperative Serum H-FABP and Age, Comorbidities and ASA Classification

A significant difference was observed in preoperative H-FABP concentrations between the two age groups. Patients aged 70 years or older had substantially higher H-FABP levels (median = 1431 ng/L, IQR = 1495) compared with those younger than 70 years (median = 550 ng/L, IQR = 890). This difference was statistically significant (U = 149.00, *p* < 0.001) with a large effect size (r = 0.574). No statistically significant differences were observed in preoperative H-FABP concentrations between patients with and without comorbidities (U = 187.00, *p* = 0.335). A statistically significant difference was observed in preoperative H-FABP concentrations between ASA 2 and ASA 3 patients. Individuals classified as ASA 3 had markedly higher H-FABP levels (median 1309 ng/L, IQR 1658) compared with those in ASA 2 (median 620 ng/L, IQR 1209), and this difference was statistically significant (U = 202.50, *p* = 0.025) with a moderate effect size (r = 0.373). ASA 3 patients had significantly higher postoperative H-FABP concentrations on POD2 (*p* = 0.004, r = 0.480) and POD4 (*p* = 0.023, r = 0.348), while no significant difference was observed on POD6 (*p* = 0.057).

### 3.5. Preoperative Serum H-FABP and Overall Postoperative Complications

Complications were observed in 38 patients (71.6%), while in the excluded cohort, 40 patients (67.8%) experienced complications. This difference was not statistically significant. Preoperative serum concentrations of H-FABP were not significantly associated with overall postoperative complications (*p* = 0.225), CR-POPF (*p* = 0.843), PPAP (*p* = 0.545), DGE (*p* = 0.192), BL (*p* = 0.758), or PPH (*p* = 0.528). Logistic regression analysis likewise demonstrated no significant association between preoperative H-FABP concentrations and postoperative complications, with odds ratios approximating unity and AUC values ranging from 0.51 to 0.61.

### 3.6. Postoperative H-FABP Dynamics in Patients with Severe Postoperative Complications

Severe postoperative complications, classified as Clavien–Dindo grade III or higher, were observed in 12 patients, accounting for 22.6% of the cohort. Postoperative serum H-FABP concentrations were significantly associated with the occurrence of severe postoperative complications. Patients who experienced severe complications exhibited notably higher H-FABP concentrations on POD2, POD4 and POD6 (Table 2).

Exploratory ROC analysis demonstrated moderate discrimination between patients with and without severe postoperative complications, with area under the curve (AUC) values as follows: POD2: AUC ≈ 0.70, POD4: AUC ≈ 0.71, and POD6: AUC ≈ 0.73. (Table 3.)

**Figure 2:**
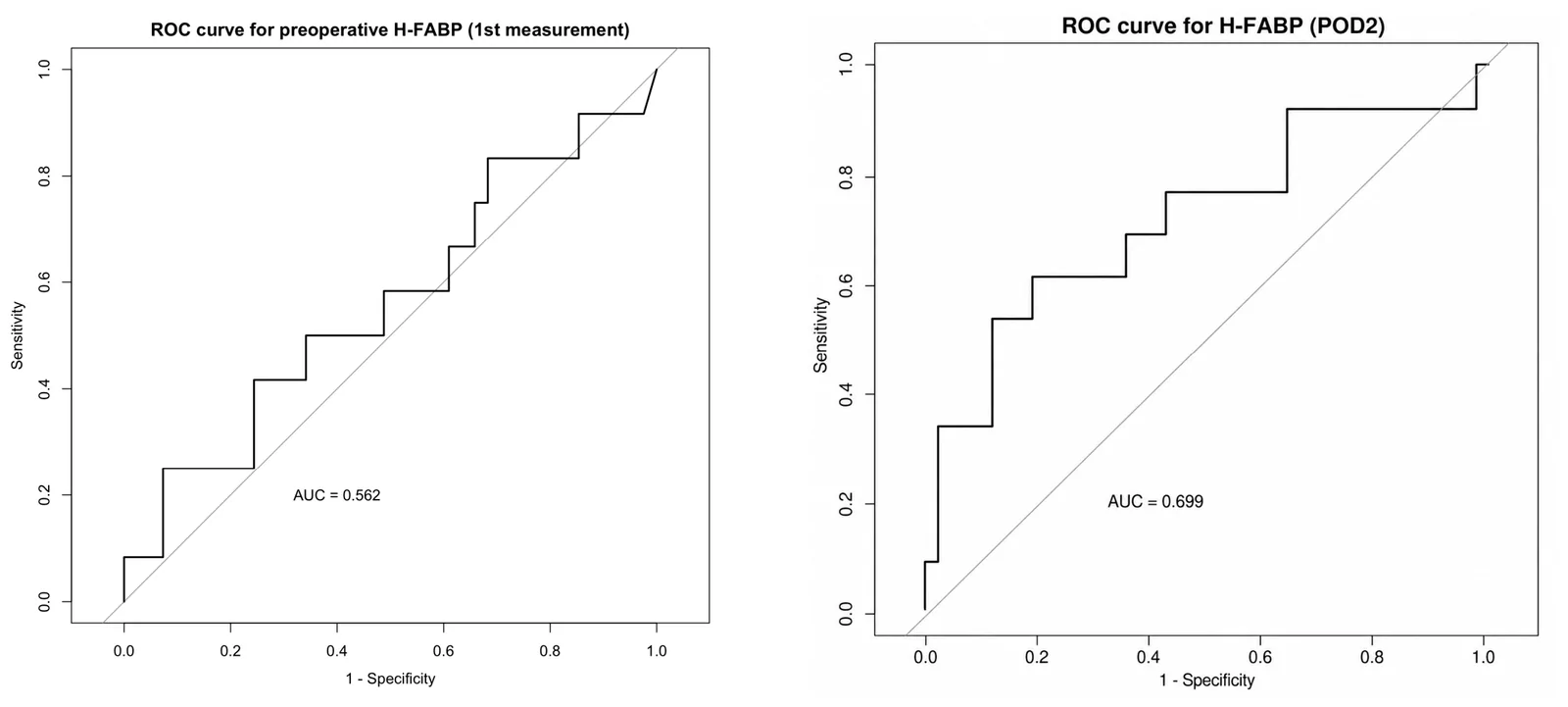

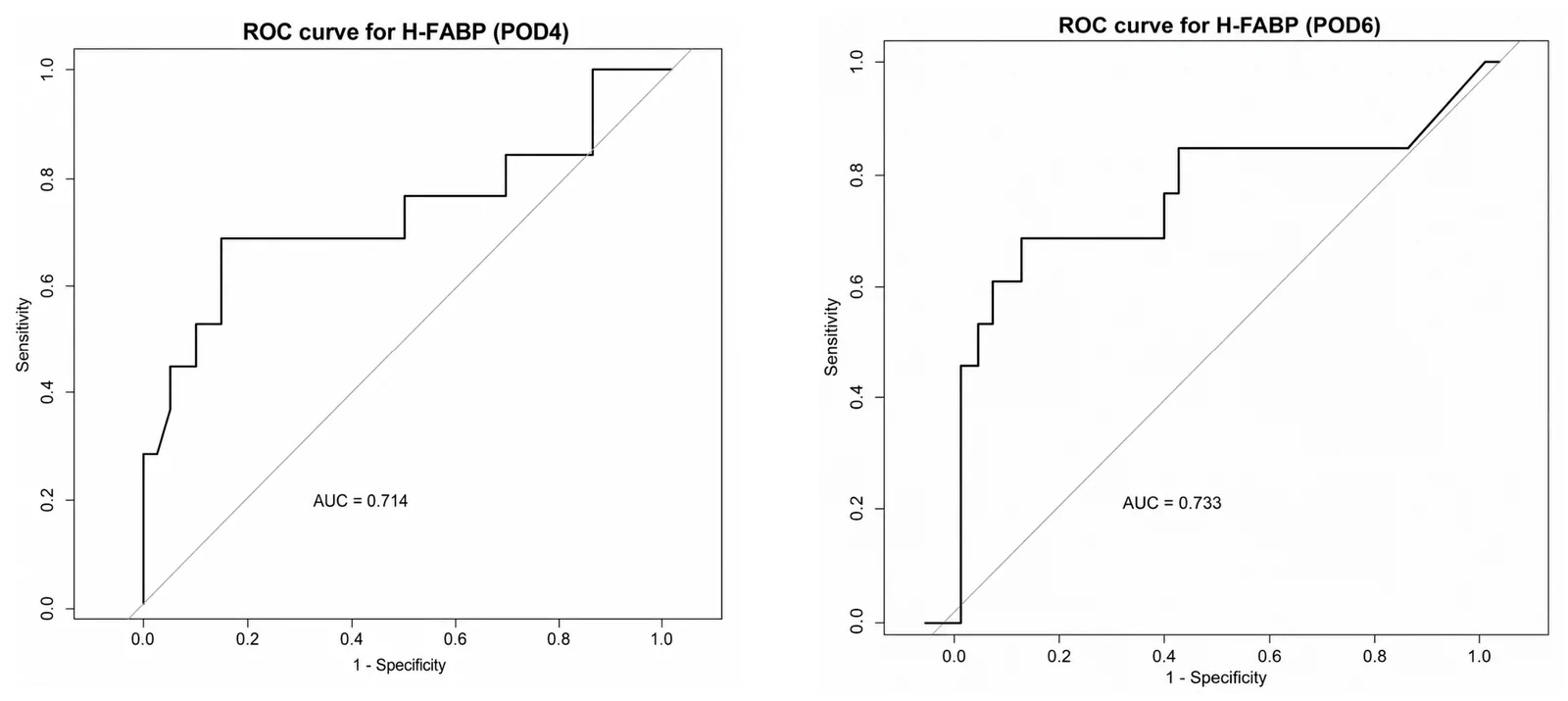
ROC curves for H-FABP measured preoperatively and on postoperative days for severe postoperative complications.

The ROC analyses, derived from a limited number of events, demonstrated only modest discrimination. The AUC values ranged from 0.70 to 0.73, accompanied by wide confidence intervals (Figure 2). Furthermore, no formal comparison between AUCs at different postoperative time points was performed. Consequently, these ROC analyses should be interpreted as preliminary results.

To account for the within-patient correlation of serial H-FABP measurements, postoperative concentrations were analyzed using a linear mixed-effects model after log(H-FABP + 1) transformation, with time, severe-complication status, and their interaction as fixed effects and patient as a random intercept. The overall between-group effect was not statistically significant (β = 0.373, 95% CI −0.832 to 1.577; *p* = 0.544). Compared with POD2, H-FABP concentrations did not differ significantly on POD4 (β = −0.367, 95% CI −1.027 to 0.294; *p* = 0.277) but were significantly lower on POD6 (β = −1.282, 95% CI −1.948 to −0.617; *p* < 0.001). No significant group-by-time interaction was observed for POD4 versus POD2 (β = 0.400, 95% CI −0.988 to 1.788; *p* = 0.572) or POD6 versus POD2 (β = 0.718, 95% CI −0.673 to 2.109; *p* = 0.312), indicating no evidence that the temporal pattern of H-FABP differed between patients with and without severe complications.

### 3.7. Postoperative Serum H-FABP Dynamics in Patients with Pancreas-Specific Complications

Pancreas-specific complications in the analyzed and excluded cohorts are presented in Table 4 and Table S1. No statistically significant differences were observed between the analyzed and excluded groups concerning pancreas-specific complications, including CR-POPF, DGE, PPH, PPAP, and BL. CR-POPF was numerically more frequent in the excluded cohort (28.8%) than in the analyzed cohort (16.9%), although this difference did not reach statistical significance (*p* = 0.17). Given the sample size, clinically relevant differences between the cohorts cannot be excluded.

Patients diagnosed with DGE exhibited significantly elevated postoperative H-FABP concentrations on POD4 (*p* = 0.010, r = 0.537) and POD6 (*p* < 0.001, r = 0.731), indicating moderate to large effect sizes. Similarly, patients experiencing BL demonstrated significantly higher postoperative H-FABP concentrations across all postoperative days, with statistically significant differences observed on POD2 (*p* = 0.005, r = 0.880), POD4 (*p* < 0.001, r = 0.952), and POD6 (*p* = 0.006, r = 0.863). In contrast, no statistically significant differences were detected in the serum concentrations of H-FABP on POD2, POD4, and POD6 among patients with CR-POPF (*p* = 0.600, *p* = 0.364, *p* = 0.293), PPAP (*p* = 0.195, *p* = 0.971, *p* = 0.236), PPH (*p* = 0.416, *p* = 0.316, *p* = 0.316). Because only three patients developed BL, these findings should be interpreted cautiously. These secondary analyses also involved multiple time-point and complication-specific comparisons without formal multiplicity adjustment and therefore should not be interpreted as confirmatory.

## 4. Discussion

The present study examined the perioperative dynamics of H-FABP in patients undergoing pancreatic resection and explored its potential association with postoperative morbidity. In this prospective exploratory study, elevated postoperative H-FABP concentrations were associated with severe postoperative morbidity after pancreatic resection, whereas preoperative concentrations were associated with higher age and ASA status.

### 4.1. Postoperative H-FABP and Systemic Surgical Stress

In our study cohort, postoperative H-FABP concentrations measured on POD2, POD4, and POD6 were significantly associated with severe postoperative complications. Because the temporal relationship between H-FABP measurement and the onset of individual complications was not prospectively established, these findings suggest associations with a complicated postoperative course rather than predictive or prognostic performance. Exploratory ROC analyses demonstrated moderate discrimination between patients with and without severe postoperative morbidity, with AUC estimates ranging from 0.70 to 0.73. However, these estimates were based on only 12 severe events and were accompanied by wide, substantially overlapping confidence intervals. No formal comparison between ROC curves was performed, and the numerical differences in AUC across postoperative time points should therefore not be interpreted as evidence of improving discrimination over time.

The biological mechanisms underlying the association between postoperative H-FABP concentrations and severe morbidity cannot be determined from the present study. One possible interpretation is that H-FABP elevation reflects the cumulative physiological burden associated with postoperative complications rather than a pancreas-specific process. However, this remains speculative. Major pancreatic surgery induces a complex response involving inflammatory activation, oxidative stress, ischemia–reperfusion phenomena, and substantial hemodynamic fluctuations [25,26,27]. These processes collectively affect myocardial oxygen supply-demand balance and cellular metabolism. H-FABP has biological characteristics that may make its circulating concentration responsive to perioperative physiological disturbances. As a small cytosolic protein involved in intracellular fatty acid transport, H-FABP is rapidly released into circulation following even subtle disruption of cardiomyocyte membrane integrity [13,14]. Plasma concentrations typically rise earlier than cardiac troponins and decline relatively quickly due to renal clearance [28]. Although H-FABP may be released in response to myocardial or other tissue injury during major surgery, its circulating concentration is also influenced by renal elimination [13,28]. Consequently, postoperative elevations may reflect increased release, impaired renal clearance, or a combination of these mechanisms. This distinction is particularly relevant following pancreatic surgery, where major complications may be accompanied by substantial physiological disturbances that can affect both tissue perfusion and renal function. Because serial creatinine, estimated glomerular filtration rate, cardiac biomarkers, and inflammatory markers were not systematically collected, we cannot attribute the observed H-FABP elevations specifically to myocardial injury or systemic stress. Accordingly, H-FABP should be regarded in the present study as a biomarker associated with a complicated postoperative course rather than as a indicator associated with specific pathophysiological process.

Importantly, the associations observed were unadjusted. Preoperative H-FABP concentrations were associated with age, ASA classification, and malignant disease in the present cohort, indicating that H-FABP concentrations may reflect underlying patient and disease characteristics in addition to postoperative processes. Additional potential confounders include renal function, cardiovascular disease, systemic inflammation, and perioperative hemodynamic disturbances. Consequently, the observed association between postoperative H-FABP concentrations and severe morbidity cannot be considered independent of potential confounding factors.

Severe postoperative morbidity was evaluated as a composite outcome based on Clavien–Dindo grade ≥ III. Although this provides an established measure of clinically relevant postoperative morbidity, it combines heterogeneous complications requiring different interventions and potentially involving different physiological responses. Consequently, the observed association with H-FABP should not be interpreted as representing a uniform biological mechanism across all severe postoperative events.

In addition to the association with overall severe morbidity, postoperative H-FABP concentrations were also significantly elevated in patients who developed DGE and BL. Delayed gastric emptying represents one of the most frequent complications following pancreaticoduodenectomy and is known to substantially prolong recovery and hospital stay [23]. The pathophysiology of DGE is complex and multifactorial. A recent comprehensive systematic review and meta-analysis identified postoperative pancreatic fistula, intra-abdominal collections, and other postoperative complications as major contributors to the development of DGE, highlighting the important role of local inflammatory and systemic physiological responses in its pathogenesis [29].

Bile leaks after pancreatic surgery are typically associated with impaired anastomotic healing, local inflammation, and infectious complications [24]. It could be hypothesized that the systemic inflammatory and hemodynamic disturbances accompanying these complications contribute to H-FABP elevation; however, this mechanism cannot be evaluated from the present data. Given the very small number of events, these findings are highly unstable and should be considered descriptive only. No conclusion regarding a specific association between H-FABP and BL or DGE can be drawn from the present cohort.

### 4.2. Preoperative H-FABP and Baseline Physiological Vulnerability

The observed association between preoperative H-FABP and age is consistent with current understanding of age-related cardiometabolic changes. Aging is associated with structural and functional myocardial alterations, including increased myocardial stiffness, microvascular dysfunction, impaired mitochondrial energetics, and reduced cardiomyocyte stress tolerance [30]. Even in the absence of clinically overt cardiovascular disease, elderly patients may exhibit subclinical myocardial strain that becomes detectable through sensitive biomarkers.

Similarly, patients with higher ASA classification demonstrated significantly elevated preoperative H-FABP levels. The ASA score is widely used as an indicator of systemic disease burden and perioperative risk [31]. The association between H-FABP and ASA classification raises the hypothesis that circulating H-FABP may be related to overall disease burden or physiological reserve; however, this interpretation remains speculative.

We did not specifically compare H-FABP concentrations according to the presence of established cardiovascular or renal disease. Only one patient in the analyzed cohort had documented stage 3 chronic kidney disease, limiting any meaningful subgroup analysis according to pre-existing renal impairment. When patients were compared according to the presence or absence of comorbidities overall, no statistically significant difference in preoperative H-FABP concentrations was observed. However, this broad comparison cannot exclude an influence of specific cardiovascular or renal conditions on H-FABP concentrations.

Preoperative H-FABP levels were also higher in patients with malignant histopathology. Cancer is increasingly recognized as a systemic metabolic disease characterized by chronic inflammation, oxidative stress, altered substrate utilization, and cachexia [32]. The higher preoperative H-FABP concentrations observed in patients with malignant disease may therefore reflect differences in underlying patient or disease characteristics; however, the mechanism cannot be determined from the present study.

Preoperative H-FABP was not associated with pancreas-specific postoperative complications such as CR-POPF, DGE, PPAP, PPH, or BL. These findings may indicate that H-FABP does not capture anatomical or technical risk factors related to pancreatic anastomosis or gland characteristics, which remain the primary drivers of pancreas-specific morbidity [2].

### 4.3. Clinical Implications

Serial postoperative H-FABP measurements may provide supplementary information regarding a complicated postoperative course. Although postoperative H-FABP showed moderate discriminatory ability, the present study was not designed to establish a clinically applicable threshold. Derivation of an optimal cut-off in this small exploratory cohort would be susceptible to overfitting and optimistic performance estimates; clinically relevant thresholds should therefore be established and validated in larger independent cohorts. H-FABP should not be regarded as a pancreas-specific predictor of complications. Rather, if validated in larger studies, it could potentially complement established clinical assessment, laboratory findings, and imaging during postoperative monitoring.

### 4.4. Limitations

Several limitations of this study should be acknowledged. The sample size was modest, and event numbers for specific complications were limited, reducing statistical power for subgroup analyses.

A major limitation is the high proportion of eligible patients who were not included in the final analysis because a complete four-sample set was unavailable. Of 112 eligible patients, only 53 (47.3%) had all four scheduled samples available for H-FABP analysis. This introduces a substantial risk of selection bias. Although no statistically significant differences were detected in the measured baseline characteristics or postoperative outcomes between the analyzed and excluded cohorts, the study was not powered to establish equivalence between these groups. It must be noted that CR-POPF was numerically more frequent among excluded patients. Although the three deaths in the excluded cohort occurred after POD6 and therefore did not directly account for incomplete sampling, other aspects of the postoperative clinical course, sample collection, or logistical factors may still have contributed to selection bias. Therefore, clinically relevant differences between included and excluded patients cannot be excluded, and the analyzed cohort may not fully represent the entire eligible population.

Only 12 patients experienced severe postoperative complications. Given this limited number of events, a multivariable model incorporating relevant potential confounders such as age, ASA classification, diagnosis, type of surgery, and renal function would have been at substantial risk of overfitting and unstable estimates. We therefore did not perform an adjusted multivariable analysis, and all reported associations should be considered unadjusted and exploratory.

Routine inflammatory and cardiac biomarkers, including CRP, procalcitonin, troponin, BNP and CK-MB, were not systematically collected at the predefined H-FABP sampling time points. Therefore, the relationship between H-FABP and established postoperative biomarkers could not be assessed. Future prospective studies should be done to compare serial H-FABP measurements with routinely used biomarkers. Patient-specific comorbidities, including heart disease and pulmonary disorders, as well as perioperative hemodynamic variables, vasopressor requirements, and transfusion data, were not prospectively collected and therefore could not be evaluated as potential confounders. Similarly, renal function parameters were not collected as part of the study protocol. Although only one patient in the analyzed cohort had documented stage 3 chronic kidney disease, transient perioperative renal dysfunction could not be assessed. Because H-FABP is primarily eliminated through the kidneys, impaired renal function may have influenced postoperative H-FABP concentrations independently of surgical stress and, together with the previously mentioned factors, represents a potential source of residual confounding.

The single-center observational design may limit the external validity and generalizability of the findings. Institutional differences in patient selection, perioperative management, surgical expertise, and postoperative care may influence postoperative H-FABP concentrations and their association with clinical outcomes. Consequently, our findings should be interpreted as hypothesis-generating and require validation in larger, multicenter prospective studies before H-FABP can be considered for routine clinical use.

## 5. Conclusions

In patients undergoing pancreatic resection, preoperative H-FABP concentrations were associated with baseline patient characteristics, whereas postoperative concentrations were associated with severe postoperative morbidity. The mechanisms underlying postoperative H-FABP elevation remain uncertain and may involve multiple factors, including tissue release and altered renal clearance. The longitudinal mixed-effects analysis did not demonstrate a significant overall between-group effect or group-by-time interaction. Given the limited number of severe events, potential selection bias, and inability to adjust for relevant confounders, these findings should be considered exploratory.

H-FABP may warrant further investigation as a complementary postoperative indicator of ongoing complication. It should be considered an adjunct too, rather than a replacement for established clinical assessment and postoperative monitoring. It could potentially contribute to multimodal postoperative monitoring aimed at detection of clinical deterioration in high-risk surgical populations, but its clinical utility requires validation in larger prospective multicenter cohorts.

## Figures and Tables

**Table 1:** Sample Characteristics.

	Analyzed Cohort (*n* = 53)	Excluded Cohort (*n* = 59)	*p*-Value
	No. (%)	No. (%)	
**ASA score**			0.84
2	19 (35.8)	23 (39%)	
3	34 (64.2)	36 (61%)	
**Age group**			0.34
<70 years	25 (47.2)	34 (57.6)	
≥70 years	28 (52.8)	25 (42.4)	
**Type of operation**			
Pancreaticoduodenectomy	29 (54.7)	29 (49.1)	0.57
Distal pancreatectomy	20 (37.7)	27 (45.8)	0.44
Other	4 (7.6)	3 (5.1)	0.70
**Aetiology**			0.17
Malignant	36 (67.9)	32 (54.2)	
Non-malignant	17 (32.1)	27 (45.8)	

**Table 2:** Comparison of postoperative H-FABP concentrations between patients with and without severe postoperative complications (Mann–Whitney U test).

Measuerment	Postoperative Day	No Severe Complications (*n* = 41) Median (IQR)	≥1 Severe Complication (*n* = 12) Median (IQR)	U	z	*p*-Value	r
**H-FABP**	POD 2	1596 ng/L (2276 ng/L)	3112 ng/L (3208 ng/L)	137.50	−2.226	0.026	0.427
	POD 4	765 ng/L (949 ng/L)	2190 ng/L (2511 ng/L)	137.50	−2.295	0.022	0.315
	POD 6	829 ng/L (913 ng/L)	2050 ng/L (1658 ng/L)	128.00	−2.437	0.015	0.466

**Table 3:** ROC analysis of H-FABP for severe postoperative complications.

Measurement (H-FABP)	AUC	95% CI		*p*-Value
		Lower	Upper	
Preoperative	0.562	0.367	0.757	0.517
POD2	0.699	0.512	0.886	0.037
POD4	0.714	0.515	0.912	0.026
POD6	0.733	0.544	0.922	0.015

**Table 4:** Overall and pancreas specific complications.

	Analyzed Cohort (*n* = 53)	Excluded Cohort (*n* = 59)	*p*-Value
	No. (%)	No. (%)	
**Overall**	38 (71.6)	40 (67.8)	0.68
**CR-POPF**	9 (16.9)	17 (28.8)	0.17
**DGE**	9 (16.9)	10 (16.9)	1.00
**PPH**	7 (13.2)	5 (8.4)	0.54
**PPAP**	3 (5.6)	7 (11.8)	0.32
**BL**	3 (5.6)	3 (5.3)	1.00

## Data Availability

The data presented in this study are available on request from the corresponding author. Data are not publicly available due to local data protection law.

## Supplementary Materials

The following supporting information can be downloaded at https://www.mdpi.com/article/10.3390/medicina62091745/s1, Table S1: Procedure specific postoperative complications.

## Appendix A. H-FABP in Pancreatic Surgery Data Procurement Protocol

### Appendix A.1. Inclusion Criteria

Patients were eligible for inclusion if they met all of the following criteria:

Aged 18 years or older

Written informed consent obtained

Scheduled for pancreatic resection for a malignant or non-malignant indication

### Appendix A.2. Exclusion Critertia

Total pancreatectomy

Exploratory nature of procedure

Incomplete four-sample set

### Appendix A.3. Preoperative Variables

**Variable**	**Recorded Data**
Patient ID	Study identification number
Age	<70 years/≥70 years
Sex	Male/Female
ASA classification	I–IV
Indication for surgery	Malignant (adenocarcinoma, neuroendocrine tumour) or non-malignant (IPMN, MCN, SCN, other)

### Appendix A.4. Intraoperative Variables

**Variable**	**Recorded Data**
Surgical approach	Open/Laparoscopic/Robotic
Procedure	Pancreaticoduodenectomy, Distal pancreatectomy, Other (Central pancreatectomy, Enucleation)

### Appendix A.5. Blood Sampling Protocol

Preoperative,

postoperative day 2, 4, 6

Serum samples were collected according to the study protocol and analyzed using ELISA.

### Appendix A.6. Postoperative Outcomes

Histopathology report: malignant/non-malignant

Procedure specific postoperative complications

**Complication**				
POPF	no	Biochemical leak	Grade B	Grade C
DGE	no	Grade A	Grade B	Grade C
PPH	no	Grade A	Grade B	Grade C
PPAP	no	Grade A	Grade B	Grade C
Bile leak	no	yes		
Postoperative pancreatic fistula (POPF), delayed gastric emptying (DGE), postpancreatectomy haemorrhage (PPH), postpancreatectomy acute pancreatitis (PPAP) were classified according to the corresponding ISGPS definitions and grading systems. Bile leak was classified according to the International Study Group of Liver Surgery definition.				

### Appendix A.7. Overall Postoperative Outcomes

Highest Clavien-Dindo grade:

▪ Grade 1
▪ Grade 2
▪ Grade 3a
▪ Grade 3b
▪ Grade 4a
▪ Grade 4b
▪ Grade 5

90-day mortality

- Yes
- No

If yes: Date: ______________ Cause of death: ________________
