# Supplementary material for: Perioperative H-FABP and Its Association with Postoperative Morbidity Following Pancreatic Resection: A Prospective Observational Study
Source: Medicina (Kaunas). 2026 Sep 10;62(9):1745. doi: 10.3390/medicina62091745 (PMC13609180; doi:10.3390/medicina62091745)
Supplement: Supplementary file 1 [file medicina-62-01745-s001.zip › medicina-4525257-supplementary.pdf]

Supplementary Table S1. Procedure specific postoperative complications.

Patient	Highest Clavien-Dindo	Complication	POD of intervention	Type of Intervention
1	IIIb	Bleeding	1	Haemostasis on PJA
2	IVb	Bleeding, POPF	2	Operative lavage, transfer to ICU due to aspiration and MOF
3	IVb	Bleeding, POPF	3	Completion pancreatectomy, transfer to ICU due to MOF
4	IIIb	POPF	3	Operative lavage
5	IIIb	BL	4	Reformation of HJA
6	IIIb	Bleeding	6	Haemostasis
7	IIIb	BL	7	Reformation of HJA
8	IIIb	Bleeding	7	Haemostasis
9	IIIb	POPF	9	Completion pancreatectomy
10	IIIb	POPF	9	Operative lavage
11	IIIa	POPF	13	Radiological drainage
12	IIIb	Laparotomy dehiscence	14	Operation
